# A multicenter case-control study on postoperative intestinal fistula in Chinese patients with Crohn disease

**DOI:** 10.1097/MD.0000000000036159

**Published:** 2023-12-08

**Authors:** Liu Zhongcheng, Yang Qingfan, Fu Xiuling, Long Meichun, Peng Bo, Xiao Zhiming, Guo Qin

**Affiliations:** a Department of Small Bowel Intestinal, The Sixth Affiliated Hospital, Sun Yat-sen University, Guangzhou, PR China; b Department of Gastroenterology, The Third Affiliated Xiangya Hospital, Central South University, Chang Sha, PR China; c Department of Gastroenterology, The Sixth Affiliated Hospital, Sun Yat-sen University, Guangdong Provincial Key Laboratory of Colorectal and Pelvic Floor Diseases, The Sixth Affiliated Hospital, Sun Yat-sen University, Guangzhou, PR China; d Department of Gastroenterology, The First Affiliated Hospital of Jishou University, Jishou, PR China.

**Keywords:** Chinese patients, Crohn disease, nomogram, postoperative intestinal fistula

## Abstract

The aim of this study was to (1) assess the independent factors affecting patients with postoperative intestinal fistula from Crohn disease (CD) by analyzing preoperative clinical data, (2) establish a nomogram prediction model for this condition based on these factors, and (3) validate this model and evaluate its accuracy. In this retrospective multicenter case-control study, the clinical data of 240 patients with CD admitted for surgical treatment between September 2019 and September 2021 at 3 centers were collected. Patients were randomly divided into a training set (168 patients) and a validation set (72 patients). Univariate analysis was performed for relevant factors, and statistically significant factors were then analyzed using multivariate logistic regression to determine the independent influencing factors. A nomogram model for predicting postoperative intestinal fistula in patients with CD was constructed and the accuracy of the model was evaluated using calibration curves. Univariate analysis showed that disease behavior, abdominal abscess, intestinal perforation, neutrophil-to-lymphocyte ratio, systemic immunoinflammatory index, and prognostic nutrition index were factors affecting postoperative intestinal fistula in patients with CD. Multivariate logistic regression analysis showed that neutrophil-to-lymphocyte ratio, prognostic nutrition index, disease behavior, and Crohn disease activity index score were independent influencing factors. After assessing the validation set, the area under the curve was 0.899, indicating good predictive accuracy of the nomogram model. The prediction model developed in this study can effectively predict the risk of postoperative intestinal fistula.

## 1. Introduction

Crohn disease (CD) is a chronic nonspecific inflammatory disease of the bowel. Despite being a major treatment modality, surgery for CD is associated with more numerous and severe postoperative complications (anastomotic fistula, intestinal fistula, multisite infection, bleeding, and short bowel syndrome) than that for other gastrointestinal diseases. These complications affect the quality of life of patients, prolong the duration of hospitalization, and lead to reoperation.^[1]^ Among them, intestinal fistula is a serious complication and a key factor leading to surgical failure that can be life-threatening if not treated properly. Thus, we retrospectively analyzed clinical data from patients with CD admitted for surgical treatment to investigate which preoperative clinical characteristics of patients were predictive of postoperative intestinal fistula. In addition, the risk factors for postoperative intestinal fistula in patients with CD were analyzed by univariate and multivariate logistic regression. A nomogram prediction model was also constructed to facilitate aggressive intervention against high-risk factors to reduce the incidence of postoperative intestinal fistula in patients with CD. This can enable patients to obtain greater benefits from treatment and provide a reference for predicting postoperative intestinal fistula.

## 2. Materials and methods

### 2.1. General characteristics

A retrospective multicenter case-control study design was used. The clinical data of patients with CD who underwent surgical treatment between September 2019 and September 2021 at the Third Xiangya Hospital of Central South University, Xiangya Hospital of Central South University, and the Sixth Affiliated Hospital of Sun Yat-sen University were collected. In total, 240 patients were included. The diagnostic criteria established by the European Crohn’s and Colitis Organisation were used for diagnosing CD^[2]^ and involved a combination of clinical manifestations and endoscopic, pathological, and imaging findings from patients. Medical record systems were used to retrieve the data of the 240 hospitalized patients. The patients were randomly divided into a training (168 patients [70%]) and a validation set (72 patients [30%]).

### 2.2. Ethics

The study was approved by the ethics committee of the Sixth Affiliated Hospital, Sun Yat-Sen University (Ethics Number: 2023ZSLYEC-176).

### 2.3. Inclusion and exclusion criteria

Inclusion criteria were as follows: (1) confirmed diagnosis of CD, (2) initial bowel resection, and (3) postoperative follow-up period of 90 days. Exclusion criteria were: (1) having only undergone strictureplasty, (2) having only undergone small bowel stoma or colostomy, (3) having only undergone perianal abscess or anal fistula surgery, (4) dying during the perioperative period, and (5) having an anastomotic fistula at postoperative follow-up.

### 2.4. Follow-up and study endpoints

The date of initial surgery for patients with CD between September 2019 and September 2021 was used as the starting point of the study. The follow-up period was 90 days. Development of intestinal fistula within or at 90 days after surgery was used as the study endpoint. Follow-up was conducted by outpatient visits, telephone inquiries, or hospitalization.

### 2.5. Diagnosis of postoperative intestinal fistula

A postoperative intestinal fistula means that the patient developed an enterocutaneous fistula within 90 days after surgery. The diagnosis was confirmed by a combination of endoscopy, imaging (e.g., computed tomography, ultrasound, magnetic resonance imaging), and subsequent laparotomy findings.

### 2.6. Study indicators

The required clinical information was ascertained through the medical record system in accordance with the inclusion and exclusion criteria. The main components included:

1. Baseline demographic information: sex, age, body mass index (BMI).
2. Baseline disease information: disease duration (length of time from diagnosis of CD to first surgical treatment), clinical presentation, disease behavior, disease site, extra-intestinal manifestations, perianal disease, and complications.
3. Disease assessment data: Crohn’s disease activity index (CDAI) score.
4. History of appendectomy.
5. Preoperative medication use: 5-mesalazine-class drugs, hormonal drugs, immunosuppressants, infliximab.
6. Laboratory indicators: complete blood count, C-reactive protein, erythrocyte sedimentation rate, albumin (ALB), globulin, D-dimer, Epstein–Barr virus (EBV) DNA or anti-EBV IgM, cytomegalovirus DNA, *Clostridium difficile*, neutrophil-to-lymphocyte ratio (NLR), platelet-to-lymphocyte ratio (PLR), C-reactive protein-to-ALB ratio (CAR), systemic immune-inflammatory index (SII, platelet count × neutrophil count/lymphocyte count), and prognostic nutrition index (PNI, serum ALB level (g/L) + 5 × total lymphocyte count).
7. Surgical status: emergency or elective surgery, open or laparoscopic surgery, type of anastomosis.

### 2.7. Statistical analysis

SPSS 22.0 software was used for statistical analysis. A random number table was used to randomly classify the patients with CD into training and validation sets. Measures approximately conforming to the normal distribution are expressed as mean ± standard deviation (*X* ± SD), and the independent-samples *t* test was used for comparison between groups. Measures not conforming to the normal distribution are expressed as median and interquartile range, and the nonparametric Mann–Whitney *U* rank-sum test was used for comparison between groups. Countable data are expressed as rates or proportions. The Pearson chi-square test was used for comparing rates, and Fisher exact test was used for data not meeting the conditions for the Pearson chi-square test. Independent factors influencing the development of intestinal fistula were first analyzed using univariate analysis, and multivariate logistic regression was performed for independent variables with *P* < .05 in univariate analysis.

R 3.5.1 and R studio were used for data analysis and nomogram plotting. A nomogram model for predicting postoperative intestinal fistula in patients with CD was constructed. Receiver operating characteristic curves were plotted to evaluate the predictive model, and the area under the curve (AUC) values were calculated. The accuracy of the model was evaluated using calibration curves, and the predictive ability of the model was validated using the validation set. Differences with *P* < .05 were considered statistically significant.

## 3. Results

### 3.1. General characteristics of patients in the training set

A total of 240 patients with CD were included in this study. Among them, 168 (70%) were in the training set, and 72 (30%) were in the validation set. Of the 168 patients with CD, 139 (82.73%) were male, and 29 (17.27%) were female. The average disease duration was 7 months (range: 1–36 months) from the diagnosis of CD to the first surgical treatment. The mean BMI was 21.32 ± 5.24 kg/m^2^. There were 46 cases of postoperative intestinal fistula (39 males and 7 females, male-to-female ratio: 5.57:1). There were 122 patients without postoperative intestinal fistula (100 males and 22 females, male-to-female ratio: 4.55:1).

### 3.2. Univariate analysis of factors influencing postoperative intestinal fistula in the training set

Univariate analysis showed that disease site, disease behavior, disease activity, abdominal abscess, intestinal perforation, intestinal obstruction, hormonal drug therapy, infliximab therapy, BMI, white blood cell count, platelet, neutrophils, lymphocyte, C-reactive protein, erythrocyte sedimentation rate, ALB, NLR, PLR, CAR, SII, and PNI were factors influencing the development of postoperative intestinal fistula in patients with CD (*P* < .05) (Tables 1–3). In contrast, sex, age of onset, duration of disease, history of appendectomy, perianal lesions, 5-mesalazine treatment, immunosuppressants, hemoglobin, hematocrit, eosinophil count, basophil count, globulin, D-dimer, emergency or elective surgery, open or laparoscopic surgery, type of anastomosis, EBV infection, cytomegalovirus infection, and *C difficile* infection were not factors that influenced the development of postoperative intestinal fistula in patients with CD (*P* > .05) (Tables 1 and 2).

**Table 1:** Univariate analysis of factors influencing postoperative intestinal fistula in patients with Crohn disease (categorical variables).

Influencing factor	Cases	Cases of complications	*X*^2^ value	*P* value
Sex			0.185	.667
M	139	39		
F	29	7		
Age at onset			1.692	.429
A1	10	3		
A2	109	33		
A3	49	10		
Site of disease			–	<.001
L1	25	4		
L2	29	6		
L3	73	33		
L4	10	0		
L1 + L4	25	0		
L3 + L4	6	3		
Disease behavior			18.508	<.001
B1	69	8		
B2	62	28		
B3	37	10		
Preoperative CDAI			28.202	<.001
Remission	54	3		
Mildly active	82	26		
Moderately active	23	10		
Severe disease	9	7		
History of appendectomy			1.584	.208
Y	30	11		
N	138	35		
Preoperative abdominal abscess			5.602	.018
Y	23	11		
N	145	35		
Preoperative perianal lesions			2.454	.117
Y	31	12		
N	137	34		
Preoperative intestinal perforation			5.300	.021
Y	54	21		
N	114	25		
Preoperative intestinal obstruction			4.631	.031
Y	76	27		
N	92	19		
Emergency surgery			3.591	.058
Y	51	19		
N	117	27		
Surgical procedure			2.033	.154
Laparoscopy	62	13		
Open	106	33		
Type of anastomosis			4.172	.243
Side-to-side	74	19		
End-to-side	61	14		
End-to-end	21	7		
Stoma	12	6		
Preoperative 5-ASA use			1.709	.191
Y	37	7		
N	131	39		
Preoperative immunosuppressant use			0.077	.781
Y	39	10		
N	129	36		
Preoperative hormone use			4.944	.026
Y	21	10		
N	147	36		
Preoperative infliximab use			3.444	.032
Y	34	5		
N	134	41		
Preoperative CMV infection ^a^			–	.319
Y	3	1		
N	73	8		
Preoperative EBV infection ^a^			–	.174
Y	11	3		
N	61	7		
Preoperative *C difficile* infection ^a^			–	1.000
Y	5	0		
N	24	3		

Note: data missing for some patients. “–” indicates Fisher exact test.

ASA = mesalazine, CDAI = Crohn’s disease activity index, CMV = cytomegalovirus, EBV = Epstein–Barr virus.

**Table 2:** Univariate analysis of factors influencing postoperative intestinal fistula in patients with Crohn disease (continuous variables).

Influencing factor	Fistula group	Non-fistula group	*t*/*Z* value	*P* value
Duration of disease (mo)	6.5 (1–36)	7 (1–36)	−0.353	.724
BMI (kg/m^2^)	19.34 ± 3.07	22.06 ± 5.69	3.970	<.001
HB (g/L)	110.63 ± 21.22	113.47 ± 25.16	0.680	.497
WBC (×109/L)	8.16 ± 2.91	7.16 ± 2.73	−2.089	.038
PLT (×10^9^/L)	306.24 ± 89.85	266.35 ± 92.17	−2.518	.013
HCT (%)	34.30 ± 8.63	36.53 ± 6.43	1.109	.270
NEU (×10^9^/L)	4.68 (3.4–6.43)	6 (4.7–8.1)	−3.034	.002
LYM (×10^9^/L)	0.82 (0.39–1.12)	1.04 (0.74–1.4)	−2.419	.033
Eosinophil count (×10^9^/L)	0.04 (0.02–0.09)	0.08 (0.02–0.12)	−1.255	.209
Basophil count (×10^9^/L)	0.01 (0.01–0.02)	0.02 (0.01–0.04)	−1.800	.072
CRP (mg/L)	39.57 (15–69.5)	14.68 (7.19–40.85)	−3.533	<.001
ESR (mm/h)	34 (20–50)	20 (10–48)	−2.216	.027
Alb (g/L)	33.08 ± 5.51	35.8 ± 5.17	2.991	.003
Globulin (g/L)	25.46 ± 5.01	25.54 ± 6.08	0.047	.963
D-dimer (mg/L)	0.64 (0.5–1.36)	0.49 (0.34–1.02)	−1.168	.243

Alb = albumin, BMI = body mass index, CRP = C-reactive protein, ESR = erythrocyte sedimentation rate, HB = hemoglobin, HCT = hematocrit, LYM = lymphocyte, NEU = neutrophils, PLT = platelet, WBC = white blood cell.

**Table 3:** Univariate analysis of factors influencing intestinal fistula after Crohn disease surgery (count data of complex hematological indicators).

Influencing factor	Intestinal fistula group	Non-intestinal fistula group	*t*/*Z* value	*P* value
NLR	6.97 (5.15–11.66)	4.2 (2.8–7.33)	−4.800	<.001
PLR	378.8 (263.64–623.08)	246.44 (163.7–364.89)	−4.073	<.001
CAR	1.14 (0.52–2.31)	0.42 (0.21–0.99)	−3.782	<.001
SII	2168.14 (1399.08–3659.43)	1068.34 (682.11–2104.14)	−5.005	<.001
PNI	37.43 ± 5.75	41.5 ± 6.18	3.879	<.001

CAR = C-reactive protein-to-albumin ratio, NLR = neutrophil-to-lymphocyte ratio, PLR = platelet-to-lymphocyte ratio, PNI = prognostic nutrition index, SII = systemic immune-inflammatory index.

**Table 4:** Multivariate analysis of factors influencing postoperative intestinal fistula in patients with Crohn disease.

	*B*	Standard error	Wald	Sig.	OR	95% CI for OR	
						Lower	Upper
NLR	0.078	0.029	7.545	0.006	1.082	1.023	1.144
PNI	−0.108	0.043	6.417	0.011	0.898	0.826	0.976
B							
1	Reference		11.523	0.003			
2	1.841	0.542	11.522	0.001	6.305	2.178	18.259
3	1.169	0.642	3.312	0.069	3.219	0.914	11.336
CDAI score							
Remission	Reference		13.940	0.003			
Mildly active	2.122	0.707	9.003	0.003	8.344	2.087	33.358
Moderately active	2.434	0.805	9.140	0.003	11.408	2.354	55.284
Severe disease	3.522	1.085	10.543	0.001	33.840	4.039	283.541
Constant	−0.301	1.754	0.029	0.864	0.740		

2, disease behavior. CDAI = Crohn’s disease activity index, CI = confidence interval, NLR = neutrophil-to-lymphocyte ratio, OR = odds ratio, PNI = prognostic nutrition index.

### 3.3. Multivariate analysis of factors influencing postoperative intestinal fistula in the training set

Multivariate logistic regression analysis was performed using statistically significant indicators from the univariate analysis as the independent variables and the development of postoperative intestinal fistula as the dependent variable. The analysis showed that NLR, PNI, disease behavior (B2), and CDAI scores were independent factors influencing the development of postoperative intestinal fistula (Table 4). The results showed that B2 (*P* = .001, odds ratio (OR) = 6.305, 95% confidence interval (CI): 2.178–18.259), high NLR (*P = *.006, OR = 1.082, 95% CI: 1.023–1.144), and the CDAI scores [mildly active (*P* = .003, OR = 8.344, 95% CI: 2.087–33.358), moderately active (*P = *.003, OR = 11.408, 95% CI: 2.354–55.284), and severely active (*P = *.001, OR = 33.840, 95% CI: 4.039–283.541)] were risk factors for postoperative intestinal fistula in patients with CD, whereas high PNI (*P = *.011, OR = 0.898, 95% CI: 0.826–0.976) was a protective factor.

### 3.4. Multivariate logistic regression equation construction

A regression equation was constructed with the development of postoperative intestinal fistula as the dependent variable and NLR, PNI, B2, and CDAI score as the independent variables. The equation is as follows:


$$\begin{array}{l} \begin{array}{lllll} & Logit\;\left(\;p\right)=-0.301+0.078\times NLR-0.108\times PNI\; & \;\;\;\;\;\;\;\;\;\;\;\;\;\;\;\;\;\;\;\;\;+1.841\left(B2\right)+2.122\;\left(mildly\;active\;period\right)\; & \;\;\;\;\;\;\;\;\;\;\;\;\;\;\;\;\;\;\;\;\;+2.434\;\left(moderately\;active\;period\right)\; & \;\;\;\;\;\;\;\;\;\;\;\;\;\;\;\;\;\;\;\;\;+3.522\;\left(severely\;active\;period\right),\; \end{array} \end{array}$$

where *p* is the probability of postoperative intestinal fistula, with values closer to 1 indicating a greater likelihood of the patient developing postoperative intestinal fistula, and values closer to 0 indicating a lower likelihood.

### 3.5. Nomogram for disease risk prediction

**Figure 1.**
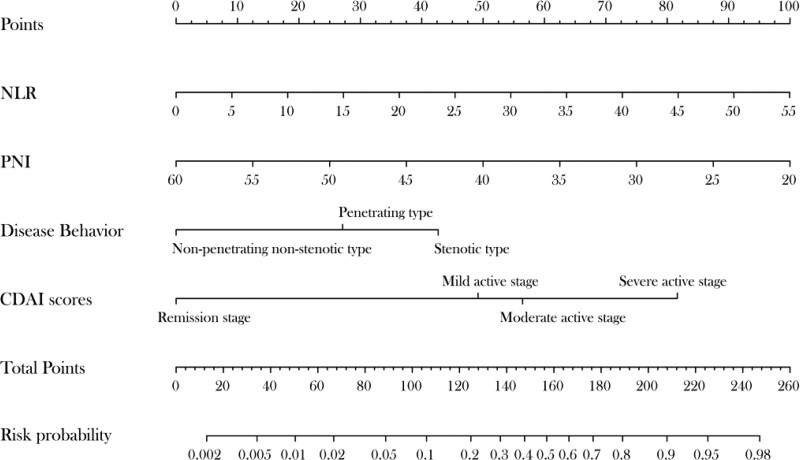
Nomogram predictive model for postoperative intestinal fistula in patients with Crohn disease. CDAI = Crohn’s disease activity index, NLR = neutrophil-to-lymphocyte ratio, PNI = prognostic nutrition index.

The independent risk factors with *P* < .05 associated with postoperative intestinal fistula based on multivariate logistic regression analysis were NLR, PNI, disease behavior (B2), and CDAI scores. These were incorporated into further model construction to construct a nomogram for calculating the risk from each factor (Fig. 1). Based on the clinical characteristics of patients with CD, a line perpendicular to the scale was made at the scores of each risk factor to obtain the points for each item, with higher scores indicating a higher probability of postoperative intestinal fistula in patients with CD. The scores of each risk factor were added to obtain the total score.

### 3.6. Evaluation of the nomogram prediction model

**Figure 2.**
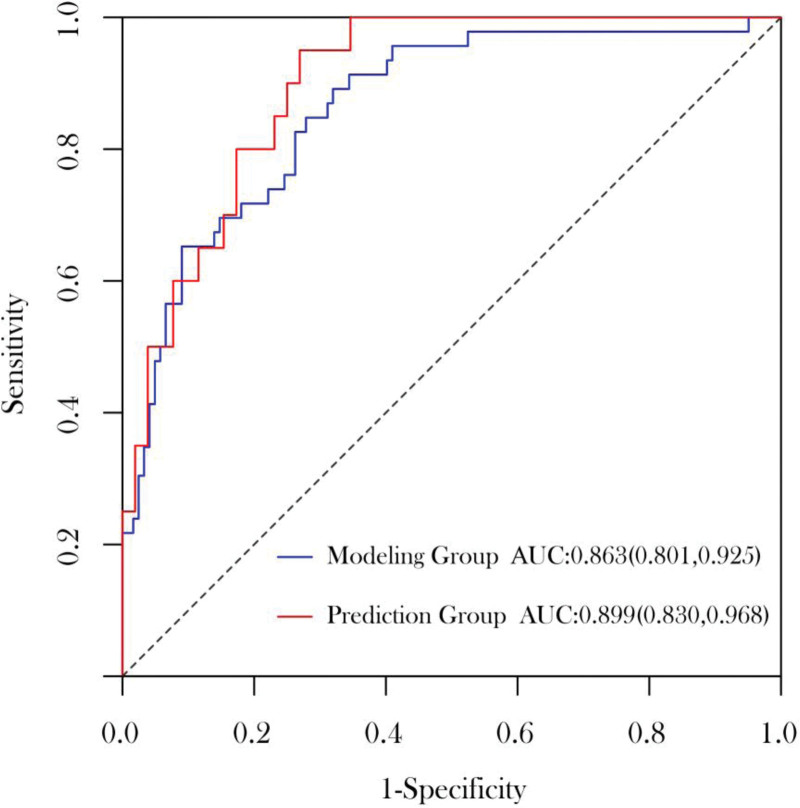
Receiver operating characteristic curve of the training and validation sets.

The prediction model was evaluated by plotting a receiver operating characteristic curve and calculating the AUC values. The AUCs of the training and validation sets were 0.863 and 0.899, respectively, suggesting good predictive ability (Fig. 2).

**Figure 3.**
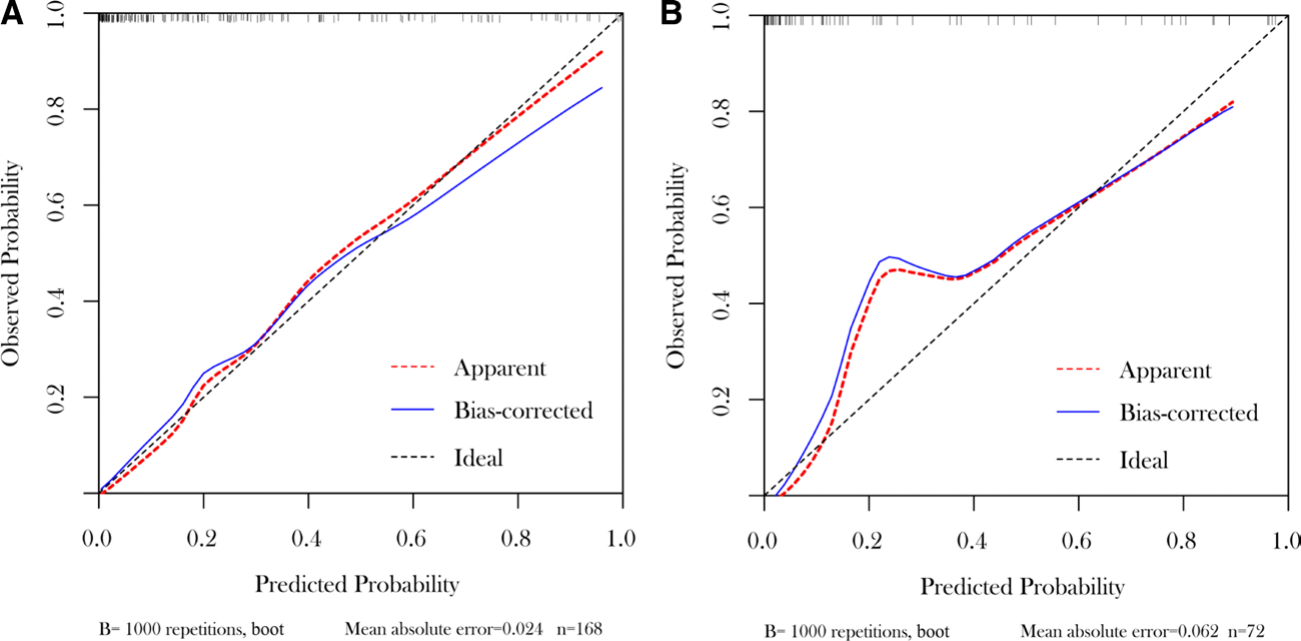
Calibration curve of the (A) modeling group and (B) prediction group.

The nomogram prediction model was validated using the validation set. The results showed a C-index of 0.899 (95% CI: 0.830–0.968, *P < *.001). Generally, a C-index between 0.50 and 0.70 indicates low accuracy, between 0.71 and 0.90 indicates moderate accuracy, and above 0.90 indicates high accuracy; thus, the nomogram has a good predictive value. Next, the calibration curves for the nomogram prediction model of the training and validation sets were plotted (Fig. 3). In addition, the Hosmer–Lemeshow test for goodness of fit showed *X*^2^ = 10.255 and *P = *.248 for the training set and *X*^2^ = 3.652 and *P = *.887 for the validation set. As *P* was >.05, there was no significant difference between the actual and calibration curves, suggesting that the model has a good predictive ability.

## 4. Discussion

Among patients with CD, 90% undergo surgery during disease progression, and 50% require a second operation. Thus, surgery remains a major treatment modality for CD.^[3]^ There is currently a lack of domestic and international multicenter clinical studies reporting on the development of enterocutaneous fistula following CD surgery with predictive models. Some single-center studies from China have reported the incidence of postoperative complications of CD to be 5.92% to 32.3%, among which the incidence of enterocutaneous fistula accounted for 8.3% to 58.6% of the total complication rate.^[4]^ Previous international studies have reported the incidence of postoperative complications of CD to be 5.0% to 40.9%, with the incidence of enterocutaneous fistula accounting for 6.0% to 76.4% of the total complication rate.^[5,6]^ Postoperative intestinal fistula can cause changes such as abdominal infection, malnutrition, and multi-organ failure. Given the severity of this complication, the prevention of postoperative intestinal fistula in patients with CD warrants attention. The findings of previous domestic and international studies indicate that emergency surgery, type of anastomosis, disease activity, increased hematocrit, preoperative anemia, preoperative malnutrition, decreased preoperative body mass, preoperative azathioprine treatment, preoperative steroid treatment, preoperative antitumor necrosis factor-α treatment, and perforation lesions were considered factors influencing the development of various complications after surgery in patients with CD. However, the results of various independent studies have not been consistent.^[7–9]^ Thus, the present study combined data from 3 inflammatory bowel disease centers in China to investigate the factors influencing the development of postoperative intestinal fistula in patients with CD and to develop a predictive model.

Various single serological indicators have low specificity and large errors in evaluating postoperative complications in patients with CD. In light of this, the aim of the present study was to evaluate disease activity and the development of postoperative complications in CD by establishing composite serologic indexes. By collecting clinical data and serological indicators of patients with CD from 3 large tertiary hospitals, it was found that NLR, PLR, CAR, SII, and PNI were all factors influencing the development of postoperative intestinal fistula. High NLR and low PNI were risk factors for postoperative intestinal fistula; the higher the NLR, the greater the risk of postoperative intestinal fistula, and the higher the PNI, the lower the risk of postoperative intestinal fistula. NLR and PLR can be determined easily from complete blood count and have been confirmed as biomarkers of inflammation and to be important in disease prognosis. Several studies have reported their correlation with the prognosis of inflammatory diseases such as acute/chronic pancreatitis; hepatitis; cancers of the gastrointestinal tract, liver, and pancreas; as well as cardiovascular disease, and it has also been used to assess CD activity.^[10]^ Inflammation and cancer have been suggested to cause tissue necrosis, which can elevate NLR and increase inflammatory mediators in the body, thereby triggering inflammatory cascades.^[11]^ Lymphocytes are major inflammatory cells of the immune system that can kill pathogenic microorganisms when inflammation is present but are also accompanied by the depletion of lymphocytes, so NLR can be used as an indicator of inflammation in determining the prognosis of patients with inflammatory diseases. PNI is an indicator that responds to both the nutritional and immune status of the body, and it is often used for determining the prognosis and severity of disease in patients with chronic diseases.^[12]^ A study showed that lower preoperative PNI levels are a marker of disease activity in patients with CD.^[13]^ Clinicians should be aware of the perioperative management of CD patients with low PNI. Therefore, the management of antibiotics and enteral and parenteral nutrition is particularly important. Moreover, it is important to optimize the implementation of prevention strategies at early stages before the operation to reduce the development of postoperative enterocutaneous fistula in patients with CD.

The results of the present study showed that the CDAI score is a factor influencing the development of postoperative enterocutaneous fistula in patients with CD. Multivariate logistic regression analysis suggested that higher disease activity resulted in a higher risk of postoperative intestinal fistula. The CDAI score can be used for assessing current disease activity in patients with CD, as well as for dynamic monitoring of changes in disease activity after CD treatment. It can also indicate the recurrence of symptoms after surgery, making it a benchmark for measuring disease severity, clinical response, and remission rates. A study showed a higher incidence of postoperative infectious complications in patients with active CD than in patients in remission.^[14]^

The present study suggested that disease behavior influences the development of postoperative intestinal fistula in patients with CD. Multivariate logistic regression analysis suggested that the risk was significantly higher in patients with stricturing CD than in patients with non-stricturing and non-perforating CD. Perforating CD was not an independent risk factor for the development of postoperative intestinal fistula. Wang et al^[15]^ included 142 Chinese patients with CD and suggested that patients with perforating CD are more likely to develop recurrence after surgery. Perforation lesions in the intestinal wall can involve adjacent organs and tissues, forming enterovesical or rectovaginal fistulas and leading to abdominal abscesses. In the present study, we found that patients with stricturing CD are more likely to develop intestinal fistulas than patients with perforating CD. This may be due to the combination of mucosal inflammation, the release of associated molecular mediators, and increased growth factor levels that promote the recruitment and proliferation of smooth muscle cells, stellate cells, and myofibroblasts in patients with CD, leading to intestinal fibrosis. In addition, intestinal stricture due to CD differs from intestinal obstruction due to other diseases, and anti-inflammatory therapy alone or small bowel endoscopic strictureplasty may not be an effective treatment. Moreover, stricture may occur in other parts of the bowel segment as the disease progresses, even after surgical removal of the strictured bowel segment.

There is a lack of domestic and international predictive models related to the development of postoperative intestinal fistula in patients with CD. Therefore, it is important to construct practical risk models with high predictive value to help stratify at-risk patients. Based on this predictive model, clinicians can identify patients at higher risk of developing enterocutaneous fistula after surgery at an early stage, which is also significant for earlier recovery after surgery. Studies have shown that nomograms have high accuracy and ease of application in predicting disease occurrence.^[16]^ The nomogram defines a score for each independent variable based on the magnitude of the regression coefficients of all independent variables. The scores of all independent variables are then added to yield a total score, which is used to calculate the probability of postoperative intestinal fistula for each patient. In the present study, 4 independent factors were identified based on multifactorial logistic regression analysis, namely disease behavior (B2), NLR, PNI, and CDAI scores. These were integrated into a nomogram model for predicting the development of postoperative intestinal fistula, with a C-index of 0.899 (95% CI: 0.830–0.968, *P < *.001) obtained after evaluation of the validation set. Finally, similar results were obtained in the Hosmer–Lemeshow goodness-of-fit test, indicating that the model has good predictive properties.

As a retrospective analysis, this study had some limitations. First, the study results may have been influenced by factors such as recall bias and follow-up timeframe. Second, the present study only observed the development of postoperative intestinal fistula, whereas the reoperation rate, hospitalization rate, and the development of other complications were not analyzed. Therefore, we expect that more data from patients with CD in inflammatory bowel disease centers and more comprehensive clinical data from patients will be collected in future prospective cohort studies to confirm the accuracy and utility of this prediction model.

In conclusion, there is a lack of domestic and international multicenter studies and nomogram models of risk factors for the development of postoperative intestinal fistula after CD. Studying the pattern of CD progression, identifying independent influencing factors for postoperative intestinal fistula in CD, and facilitating aggressive intervention against high-risk factors, thus reducing the incidence of postoperative intestinal fistula in patients with CD, enables patients to reap greater benefits from treatment. The predictive model was validated externally using the validation set to effectively predict the risk of postoperative enterocutaneous fistula, which partially informs the development and adjustment of perioperative treatment plans and management strategies for patients with CD. Individuals at higher risk of developing enterocutaneous fistula should be provided early symptomatic interventions to reduce the risk of postoperative enterocutaneous fistula, thus allowing them to derive greater benefit from treatment and reduce suffering.

## Author contributions

**Conceptualization:** Yang Qingfan, Guo Qin.

**Data curation:** Liu Zhongcheng, Yang Qingfan, Fu Xiuling, Long Meichun.

**Formal analysis:** Liu Zhongcheng, Peng Bo.

**Funding acquisition:** Guo Qin.

**Investigation:** Peng Bo, Guo Qin.

**Methodology:** Liu Zhongcheng, Fu Xiuling, Long Meichun, Xiao Zhiming.

**Supervision:** Xiao Zhiming, Guo Qin.

**Writing – original draft:** Liu Zhongcheng, Yang Qingfan.

**Writing – review & editing:** Liu Zhongcheng.
